# Impact of pulmonary rehabilitation on patients’ health care needs and asthma control: a quasi-experimental study

**DOI:** 10.1186/s12890-020-01301-9

**Published:** 2020-10-15

**Authors:** Julia Salandi, Andrea Icks, Jalal Gholami, Stefan Hummel, Konrad Schultz, Christian Apfelbacher, Aziz Sheikh, Adrian Loerbroks

**Affiliations:** 1 Institute of Occupational, Social, and Environmental Medicine, Centre for Health and Society, Medical Faculty, University of Düsseldorf, Moorenstraße 5, 40225 Düsseldorf, Germany; 2 Institute of Health Services Research and Health Economics, Centre for Health and Society, Medical Faculty, University of Düsseldorf, Moorenstraße 5, 40225 Düsseldorf, Germany; 3 Institute of Health Services Research and Health Economics, German Diabetes Center, Medical Faculty, University of Düsseldorf, Auf’m Hennekamp 65, 40225 Düsseldorf, Germany; 4 Nordseeklinik Borkum der DRV Rheinland, Bubertstraße 4, 26757 Borkum, Germany; 5 MEDIAN Klinik Heiligendamm, Kinderstrand 1, 18209 Bad Doberan, Germany; 6 Klinik Bad Reichenhall der DRV Bayern Süd, Salzburger Str. 8-11, 83435 Bad Reichenhall, Germany; 7 Institute of Social Medicine and Health Systems Research (ISMHSR), Otto von Guericke University Magdeburg, Leipziger Str. 44, 39120 Magdeburg, Germany; 8 Usher Institute, University of Edinburgh, Old Medical School, Teviot Place, Edinburgh, EH8 9AG UK

**Keywords:** Asthma, Needs, Patient-reported outcome measures, Rehabilitation

## Abstract

**Background:**

Pulmonary rehabilitation offers potential benefits to people with asthma. It is however unknown if rehabilitation favourably affects patients’ health care needs. We therefore examined if rehabilitation reduced needs and, in addition, if it improved asthma control.

**Methods:**

One hundred fifty patients with asthma were surveyed in three rehabilitation clinics at admission and at discharge. Additionally, we surveyed 78 participants with asthma twice 4 weeks apart. The latter sample (i.e. the control group) was recruited through other pathways than rehabilitation clinics. The Patient Needs in Asthma Treatment (NEAT) questionnaire and the Asthma Control Test (ACT) were completed at baseline and follow-up. Differences between baseline and follow-up and between rehabilitation and control group were examined by t-tests and chi-squared-tests. Univariate ANCOVAS were used to examine if NEAT and ACT follow-up scores differed significantly between groups. Within the rehabilitation group, linear regressions were used to examine if self-reported utilization of more interventions that addressed needs were associated with NEAT scores at follow-up.

**Results:**

At baseline, there were no differences between the rehabilitation and the control group regarding needs and asthma control. At follow-up, the rehabilitation group showed reduced needs (*t(149) = 10.33*, *p* <  0.01) and increased asthma control (*t(130) = -6.67*, *p* <  0.01), whereas members of the control group exhibited no changes. Univariate ANCOVAS showed that unmet follow-up needs (*F*(1, 212) = 36.46, *p* <  0.001) and follow-up asthma control (F(1, 195) = 6.97, *p* = 0.009) differed significantly between groups. In the rehabilitation group, self-reported utilization of more interventions was associated with reduced needs (β = 0.21; *p* = 0.03).

**Conclusions:**

This study provides preliminary evidence suggestion that pulmonary rehabilitation in adults with asthma may reduce asthma-related needs and confirms previous findings that rehabilitation may improve asthma control.

## Background

Pulmonary rehabilitation is considered a key factor in the successful long-term care of patients suffering from asthma. This holds particularly true whenever patients experience adverse physical, social or psychological consequences due to their asthma despite access to and availability of adequate outpatient medical care [1]. Pulmonary rehabilitation has been conceptualized as a set of multidisciplinary interventions to reduce symptoms, stabilize or improve patients’ health status and to increase participation in their health care planning. In this context, the main goals of pulmonary rehabilitation are to attain the best possible level of daily functioning and to enable participation in social and professional life despite the presence of a chronic disease [2]. This can be achieved by facilitating patients’ coping with and self-management of the bio-psycho-social sequels of their condition (such as anxiety or reduced physical activity), which are not effectively treated by medication [1, 2].

Guidelines on pulmonary rehabilitation [3–6] and research findings [7, 8] highlight the importance of individually tailored health care programmes for patients with asthma [9]. Essential components of pulmonary rehabilitation are the diagnosis, optimization of pharmacological treatment regimes, avoidance of allergen exposure, comprehensive patient education programs, physical training, respiratory physiotherapy and psychological interventions [10, 11]. Nonetheless, in rehabilitation practice the focus of treatment varies depending on individual therapeutic goals, which are planned and determined individually for and with every person undergoing rehabilitation [12]. In the era of precision medicine and in order to reduce the already mentioned bio-psycho-social sequeles of asthma, the effectiveness of pulmonary rehabilitation should not only be measured in terms of physiological outcomes [4, 13] or by the use of health care services [4, 14], but also by patient-reported outcomes (PROs), such as asthma-specific quality of life [4, 13, 15–17] and self-reported asthma control [17, 18]. Research on the effectiveness of pulmonary rehabilitation has so far focused mainly on people with chronic obstructive pulmonary disease (COPD), and the existing research in people with asthma is limited both in terms of medical outcomes and PROs [19].

Patient-centeredness in asthma care also includes consideration of patients’ health care needs. Addressing unmet health care needs may improve patients’ adherence and health outcomes such as asthma control [7, 20]. In this context, the term *need* should not be understood as an objective need or a need recommended by guidelines or the physician (i.e. normative need), but instead as a perceived health care need that is felt by a given patient and remains unmet (e.g. information or support needs) [21, 22]. To date, no standardized screening of health care needs is used in asthma care. However, addressing those needs could facilitate planning rehabilitation goals. Closely related to this, research has yet not examined if patients’ health care needs are being reduced during rehabilitation. To measure these needs we have previously developed and validated the so-called Patient Needs in Asthma Treatment (NEAT) questionnaire [20, 23]. The NEAT assesses needs related to knowledge regarding asthma pathophysiology, medication, and emergency plans, as well as needs to train breathing techniques or the correct use of the inhaler and communication with health care staff and family or friends. Given the close match of issues covered by both pulmonary rehabilitation and the NEAT, it seems plausible that existing health care needs as measured by the NEAT may be addressed and favourably modified during pulmonary rehabilitation. Our previous research suggested that health care needs do not change in the short-term (i.e. 4 weeks) without intervention [20]. We hypothesized for the current study that participation in a specific intervention (i.e. inpatient rehabilitation) would help to reduce patients’ health care needs. Further, we hypothesized that pulmonary rehabilitation will enhance asthma control, as suggested by prior studies [17, 18].

## Methods

In order to examine if pulmonary rehabilitation can improve patients’ health care needs and asthma control, we collected data among two samples. First, we included patients who participated in an inpatient rehabilitation with specific asthma-related interventions (rehabilitation group). Second, to facilitate a comparison, we included a control group.

### Study population

#### Sample 1 – rehabilitation group

Between August 2018 and January 2019, we carried out a survey among 150 people undergoing pulmonary rehabilitation in three rehabilitation clinics in Germany, all of which adopted a bio-psycho-social approach with individualized interventions (i.e. selection of treatment according to patients’ needs). All people were at least 18 years old, German speaking, and had physician-diagnosed asthma, but did not have COPD. Participants were recruited through attending physicians during admission and, if inclusion criteria were met, were asked to complete the survey (by paper-and-pencil) at admission (before the treatment period) and at discharge (after the treatment period, thus at the end of rehabilitation). This means that the time between baseline and follow-up differed between participants, depending on the duration of rehabilitation. All patients filled in the questionnaire at the respective rehabilitation clinic. Participants in the rehabilitation group received the usual scope of care that is common in the respective rehabilitation clinic.

#### Sample 2 – control group

Between January and July 2018, we carried out a survey among 78 adults (≥18 years) who reported physician-diagnosed asthma. Participants were recruited by various pathways, e.g. by support groups or pulmonary sport groups, and were asked to complete the questionnaire twice (either web-based or by paper-and-pencil) 4 weeks apart. The participants did not have to meet any asthma-related inclusion criteria (e.g. being stable) and could fill in the questionnaire at home. The data from this sample has already been published elsewhere [20] in the context of assessing the reproducibility of the NEAT (i.e. test-retest reliability). For the present study, that sample was considered as control group which allows for the comparison of the NEAT and Asthma Control Test (ACT) scores between patients who received a specific asthma intervention (i.e. pulmonary rehabilitation) and patients who likely did not.

### Measures

#### Patients’ health care needs

Unmet needs were captured by the NEAT questionnaire [23, 24]. This 13-item instrument measured needs by four subscales (i.e. consideration of patient expertise by physicians [4 items]; information on drug effects [3 items]; information and training related to handling of drugs [3 items]; responding to exacerbations [3 items]). Items were phrased as questions with three response options: “Yes, I would like this”, “This need has already been met”; and “No, I do not need this” [23]. In line with our previous work [20, 23–25], we coded responses as 1 = yes versus 0 = no (“This need hast already been met”/“I do not need this”). We calculated mean scores across all items with higher scores reflecting more unmet needs. Details on the NEAT’s adequate psychometric properties can be found elsewhere [20, 23].

#### Asthma control

In addition to health care needs, we assessed asthma control, measured by the well-established Asthma Control Test (ACT). The ACT is a 5-item instrument assessing asthma symptoms, use of rescue medications, and the effect of asthma on daily functioning (e.g. “In the past 4 weeks, how much of the time did your asthma keep you from getting as much done at work, school or at home?”). The ACT’s potential score ranges from 5 (very poorly controlled) to 25 (completely controlled) with higher scores indicating better control. A score of 19 or less has been defined as a cut-off score suggesting poor control [26].

#### Asthma education/training

We examined to what extent participants in the rehabilitation group received specific asthma treatment or support interventions during rehabilitation that may be assumed to exert effects on specific domains on health care needs. Therefore, we asked for asthma education and training that reflected needs, surveyed in the NEAT questionnaire. In detail, we asked rehabilitation group participants the following question: “What content was conveyed during asthma education?” Response options were based on a tick-off list, e.g. “information about how to take my asthma drugs” or “practical exercise of breathing techniques, which can help during asthma attack”. We collected this data to be able to examine if (specific) PR interventions were associated with reduced needs in a dose-response fashion.

#### Demographics

In order to describe and compare both samples appropriately, we present baseline data on gender, age, school education, reported allergy (based on a tick-off list) and follow-up time.

Whereas health care needs, asthma control and demographic information were gathered in the rehabilitation and the control group, questions on specific asthma education and training during rehabilitation were only asked in the rehabilitation group.

### Statistical analyses

Initially, comparisons between rehabilitation and control group regarding demographics as well as NEAT and ACT scores were carried out by unpaired t-tests (age, follow-up period, NEAT and ACT scores) and chi-squared-tests (gender, school education, reported allergies) to examine if the samples’ measured characteristics were sufficiently comparable.

In both samples, we examined the potential changes of the NEAT total scores and subscale-specific scores by pre-post-score comparisons and tested for statistical significance based on paired t-tests. In addition, we examined the respective potential changes of the ACT sum scores also by running paired t-tests. We hypothesized that the NEAT scores would decline (i.e. indicating reduced needs) and that the ACT scores would increase (i.e. reflecting better asthma control) during the follow-up period only in the rehabilitation, but not in the control group.

Furthermore, we ran univariate ANCOVAS with group membership (1 = rehabilitation, 2 = control) as independent variable and NEAT and ACT follow-up scores as dependent variables. To consider the potentially dissimilar demographic characteristics of the rehabilitation and the control group, we included gender, age, educational level, number of allergies, follow-up period and the respective baseline score (NEAT or ACT) as covariates.

Finally, to examine if pulmonary interventions were associated with reduced health care needs in a dose-response fashion, we ran linear regression models with the total sum score of self-reported received asthma education/training during rehabilitation that addressed needs as independent variable and the NEAT total change score as dependent variable. Furthermore, we calculated subscale-specific scores capturing the type of received asthma education/training, which reflect subscale-specific topics of the NEAT. For instance, if breathing techniques and correct use of the inhaler were taught to a given patient, a reduction of the corresponding NEAT score on the subscale *exacerbations* was expected; however, if a patient reported not to have learned about patient-physician communication, a reduction of the NEAT *patient expertise* subscale score was not to be expected. Linear regressions were controlled for gender, age, educational level, duration of stay as well as for a variable reflecting the three different rehabilitation clinics.

## Results

### Demographics

In the rehabilitation group, 178 participants completed the baseline questionnaire at admission and 166 filled in the follow-up questionnaire at discharge (response rate = 93%). We excluded an additional 16 participants either because they completed the baseline questionnaire more than 3 days after admission and had therefore possibly received some interventions prior to that or because they filled out the follow-up questionnaire more than 3 days before discharge and interventions were thus potentially still pending. The final sample for our analysis comprised thus 150 participants.

In the control group, 112 people with asthma participated in the first assessment and 78 of those provided data on both occasions (response rate = 67%).

Table 1 shows characteristics of the population of sample 1 (rehabilitation) and sample 2 (control). In both samples, more women than men were surveyed (rehabilitation: 59%, control: 83%). In the rehabilitation group, participants were, on average, in their early-to-mid-fifties and had attained more frequently intermediate levels of school education. In the control group, patients were in their mid-forties and, on average, 8 years younger than participants in the rehabilitation group. In the latter sample, participants had achieved mostly intermediate or high levels of school education. The mean follow-up period in the rehabilitation group was about 23 days (standard deviation [SD] = 5.00, Min = 17, Max = 51) and in the control group about 31 days (SD = 5.25, Min = 25, Max = 48).

**Table 1:** Characteristics of the study populations and comparison between sample 1 and 2 regarding baseline demographics and NEAT ^a^ and ACT ^b^ scores

Variables	Sample 1 –rehabilitation group ***n*** = 150	Sample 2 - control group ***n*** = 78	***p*** ^**c**^
Female Gender, *n* (%)	88 (58.67)	65 (83.33)	< 0.001
Age (years), mean (SD)	52.87 (11.50)	45.26 (12.19)	< 0.001
School education ^d^, *n* (%)			< 0.001
Low	46 (30.67)	11 (14.10)	
Middle	67 (44.67)	31 (39.74)	
High	37 (24.67)	34 (43.59)	
Any allergy ^e^, *n* (%)	117 (78.00)	70 (89.74)	0.05
NEAT ^a^ total score baseline, mean (SD)	5.73 (3.39)	5.72 (3.93)	0.99
NEAT ^a^ total score follow-up, mean (SD)	3.13 (2.94)	5.93 (3.77)	< 0.001
ACT ^b^ total score baseline, mean (SD)	17.39 (4.61)	16.99 (4.81)	0.42
ACT ^b^ total score follow-up, mean (SD)	19.55 (4.17)	17.56 (5.36)	0.01
Adequate asthma control at baseline ^f^, *n* (%)	52/139 (34.66)	28/78 (35.90)	0.94
Adequate asthma control at follow-up ^f^, *n* (%)	84/139 (56.00)	34/78 (43.59)	0.03
Follow-up period, mean days (SD)	22.83 (5.00)	30.85 (5.25)	< 0.001

^a^ *NEAT* Needs in Asthma Treatment Questionnaire (score range: 0–13)

^b^ *ACT* Asthma Control Test (score range: 5–25)

^c^ Comparison between sample 1 and 2 regarding baseline demographics and NEAT and ACT scores, carried out by unpaired t tests (age, follow-up period, NEAT and ACT scores) and chi-squared-tests (gender, school education, reported allergies)

^d^ High = general qualification for university entrance (“Abitur”) or entrance qualification limited to universities of applied sciences (“Fachhochschulreife”); intermediate = secondary school level I certificate (“Mittlere Reife”); low = secondary modern school qualification (“Haupt−/Volksschulabschluss”) or no formal school degree

^e^ Reports of allergy to either foods, pollen, dust mites, fur, insect venom, drugs or allergic contact eczema in response to cosmetics, lotions and alike

^f^ Defined as a score ≥ 20 on the Asthma Control Test

Samples differed significantly in terms of gender (58.67% vs. 83.33% female; *p* <  0.001), age (52.87 vs. 45.26 years on average; *p* <  0.001), school education (30.67% vs. 14.10% low school education; *p* <  0.001), reported allergies (78% vs. 89.74% any allergy; *p* = 0.05) and follow-up period (22.83 vs. 30.85 days on average; *p* <  0.001).

### Asthma outcomes

**Fig. 1:**
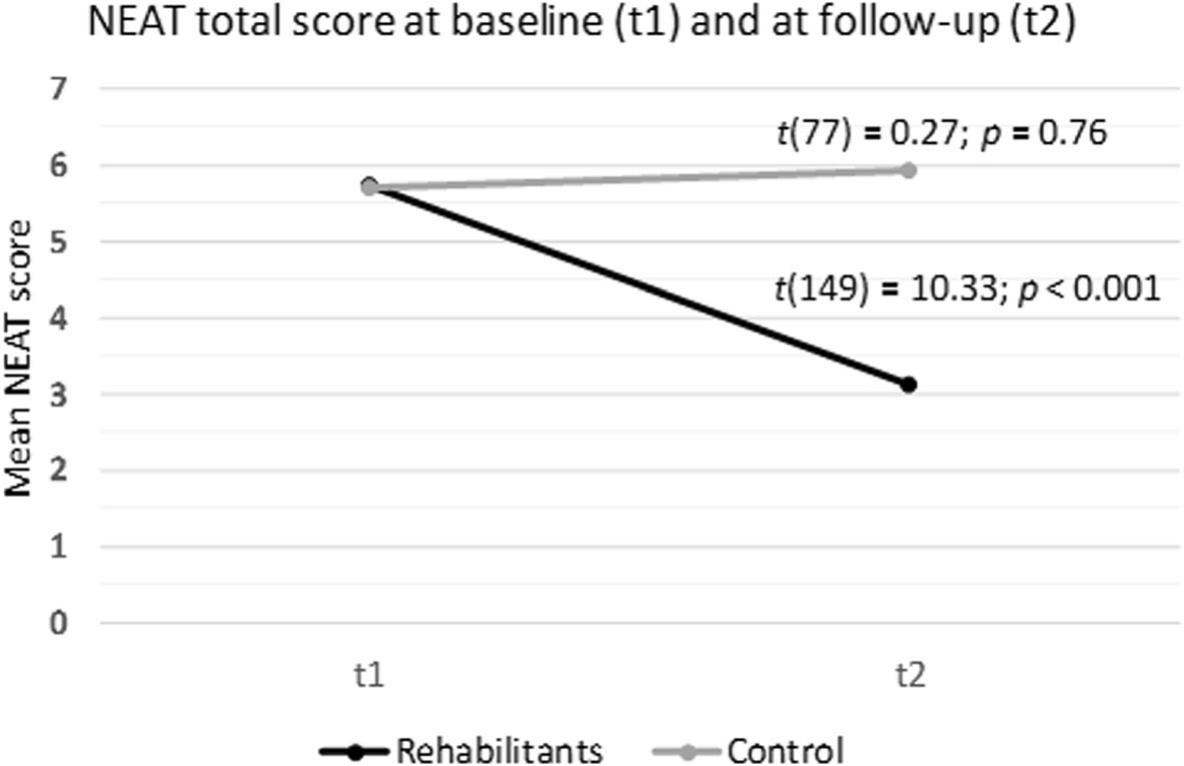
Mean change of the Patient Needs in Asthma Treatment (NEAT) questionnaire in rehabilitation and control group between baseline and follow-up

At baseline, there were no differences between both samples regarding health care needs, measured by the NEAT total mean score (5.73 in the rehabilitation vs. 5.72 in the control group). In contrast, at follow-up, we found a significantly lower NEAT total mean score in the rehabilitation as compared to the control group (3.13 in the rehabilitation vs. 5.93 in the control group; *p* <  0.01). This was explained by a significant reduction of unmet needs in the rehabilitation group (*total need score: t*(149) = 10.33; *p* <  0.001) compared to their baseline score whereas the score in the control group remained stable (Fig. 1).

**Fig. 2:**
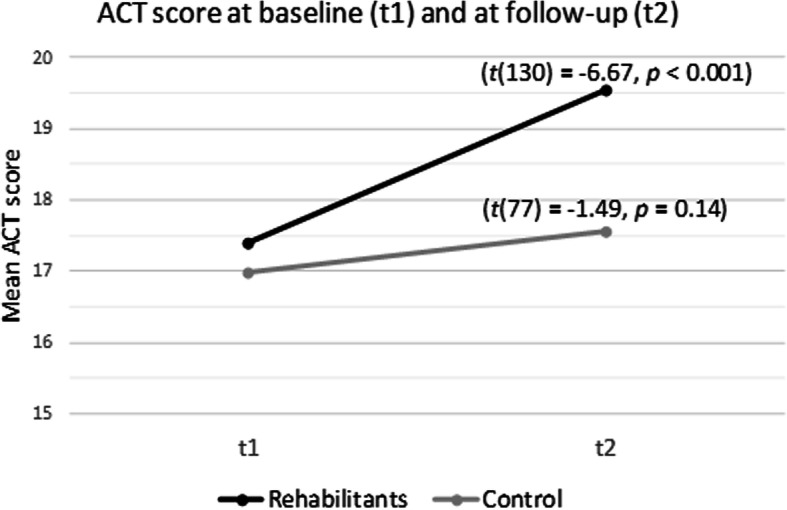
Mean change of the Asthma Control Test (ACT) in rehabilitation and control group between baseline and follow-up

Furthermore, in both samples asthma control was comparably poor at baseline in light of the fact that a score of 19 or lower defines poor control (mean score of 17.39 in the rehabilitation vs. 16.99 in the control group). In contrast, at follow-up, the rehabilitation group showed significantly higher ACT mean scores compared to the control group (19.55 in the rehabilitation vs. 17.56 in the control group, *p* = 0.01). We found that in the rehabilitation group asthma control significantly increased during follow-up period to an rather appropriate level (well controlled ≥ 20) (*t*(130) = − 6.67, *p* <  0.001), whereas in the control group asthma control remained poor and exhibited little and non-significant change (Fig. 2). Table 2 shows paired t tests of NEAT total and subscale scores and ACT sum scores at baseline and follow-up in both samples.

**Table 2:** Paired t tests of NEAT ^a^ total and subscale scores and ACT ^b^ sum scores at baseline and follow-up in both samples

Variables	T1^**c**^: mean (SD)	T2^**d**^: mean (SD)	***t***	df	***p***
**Rehabilitation group**					
**NEAT** ^**a**^ **total score**	0.46 (0.27)	0.24 (0.23)	10.33	149	< 0.001
**Patient expertise** ^**e**^	0.44 (0.37)	0.29 (0.33)	4.77	132	< 0.001
**Drug effects** ^**e**^	0.65 (0.36)	0.37 (0.39)	8.16	145	< 0.001
**Handling drugs** ^**e**^	0.19 (0.30)	0.08 (0.20)	3.97	142	0.006
**Exacerbations** ^**e**^	0.55 (0.39)	0.21 (0.30)	11.17	142	< 0.001
**ACT** ^**b**^ **sum score**	17.39 (4.61)	19.68 (4.12)	−6.67	130	< 0.001
**Control Group**					
**NEAT** ^**a**^ **total score**	0.44 (0.30)	0.43 (0.29)	0.27	77	0.76
**Patient expertise** ^e^	0.47 (0.39)	0.45 (0.37)	0.59	75	0.56
**Drug effects** ^**e**^	0.57 (0.37)	0.54 (0.38)	0.79	77	0.44
**Handling drugs** ^**e**^	0.23 (0.32)	0.23 (0.33)	0.00	74	1.00
**Exacerbations** ^e^	0.48 (0.41)	0.51 (0.40)	−0.97	77	0.33
**ACT** ^**b**^ **sum score**	16.99 (4.81)	17.56 (5.36)	−1.485	77	0.14

^a^ *NEAT* Needs in Asthma Treatment Questionnaire

^b^ *ACT* Asthma Control Test

^c^ *T1* assessment 1 (baseline)

^d^ *T2* assessment 2 (follow-up)

^e^ NEAT subscales

### Univariate ANCOVAS

Univariate ANCOVAS (Table 3) showed that unmet needs at follow-up (*F*(1, 212) = 36.46, *p* <  0.001), and the level of asthma control at follow-up (*F*(1, 195) = 6.97, *p* = 0.009), differed significantly between the two groups (1 = rehabilitation, 2 = control). These observations imply that the rehabilitation group showed significantly less health care needs and better asthma control at follow-up (and after pulmonary rehabilitation) compared to the control group.

**Table 3:** Univariate ANCOVAS with group membership (rehabilitation or control) as independent variable, NEAT ^a^ and ACT ^b^ follow-up scores as dependent variables and sex, age, educational level, numbers of allergies, follow-up period and the respective baseline score as covariates

Variables	Mean – Follow-up (95% CI ^**c**^)		Mean difference – Follow-up (95% CI ^**c**^)	***p*** value for difference
	Rehabilitation (***n*** = 141)	Control (n = 73)		
**NEAT** ^**a**^	0.25 (0.21–0.28)	0.44 (0.38–0.51)	− 0.19 (− 0.26 - -0.12)	< 0 .001
	**Rehabilitation** **(*n*** **= 124)**	**Control** **(*n*** **= 73)**		
**ACT** ^**b**^	19.60 (18.88–20.29)	17.48 (16.24–18.65)	2.12 (0.79–3.42)	0.009

^a^ *NEAT* Needs in Asthma Treatment Questionnaire

^b^ *ACT* Asthma Control Test

^c^ *CI* confidence interval

### Linear regression analysis

In the rehabilitation group we ran additional linear regression models to examine if self-reported utilization of more interventions that address needs, surveyed in the NEAT questionnaire, were associated with reduced health care needs in a dose-response fashion (Table 4). We found that a total score of those interventions during pulmonary rehabilitation was significantly associated with reduced needs, as measured by the NEAT total change score (β = 0.21; *p* = 0.03) as well as measured by the change scores of the NEAT subscales *patient expertise* (β = 0.31; *p* = 0.009) and *exacerbations* (β = 0.20; *p* = 0.04). Further, received interventions that addressed patient expertise were significantly associated with reduced needs, measured by the change score of the NEAT subscale *patient expertise* (β = 0.25; *p* = 0.01) and received interventions addressing exacerbations tended to be significantly associated with reduced needs, measured by the change score of the NEAT subscale *exacerbations* (β = 0.20; *p* = 0.052). In contrast, there was no evidence of associations between received interventions addressing drug effects and the change score of the NEAT subscale *drug effects* as well as between experienced interventions addressing how to handle drugs and the change score of the NEAT subscale *handling drugs*.

**Table 4:** Sample 1: Linear regression with achieved asthma treatment total and subscale scores as independent variables and NEAT ^a^ total and subscale change scores as dependent variables

Variables	NEAT ^**a**^ total change score ^**b**^			Patient expertise change score ^**b c**^			Drug effects change score ^**b c**^			Handling Drugs change score ^**b c**^			Exacerbations change score ^**b c**^		
	b	β	***p***	b	β	***p***	b	β	***p***	b	β	***p***	b	β	***p***
**Asthma treatment total score**	0.13	0.21	0.03	0.03	0.31	0.009	0.02	0.15	0.13	0.01	0.05	0.60	0.02	0.20	0.04
**Treatment in patient expertise**^**c**^	0.01	0.08	0.37	0.06	0.25	0.01	0.02	0.10	0.25	0.02	0.08	0.42	0.00	0.02	0.86
**Treatment in drug effects**^**c**^	0.04	0.15	0.08	0.04	0.12	0.20	0.06	0.14	0.12	0.00	0.01	0.91	0.07	0.18	0.04
**Treatment in handling drugs**^**c**^	0.02	0.11	0.21	0.06	0.23	0.01	−0.02	−0.07	0.47	−0.00	−0.01	0.96	0.04	0.16	0.09
**Treatment in exacerbations**^**c**^	0.05	0.20	0.06	0.04	0.12	0.27	0.09	0.24	0.02	0.02	0.06	0.60	0.07	0.20	0.05

^a^ *NEAT* Needs in Asthma Treatment Questionnaire

^b^ Adjusted for sex, age, educational level and rehabilitation clinic

^c^ NEAT subscales

Furthermore, received interventions addressing education in handling drugs were significantly associated with reduced needs, measured by the change score of the NEAT subscale *patient expertise* (β = 0.23; *p* = 0.01). Also, received interventions addressing exacerbations were significantly associated with reduced needs, measured by the change score of the NEAT subscale *drug effects* (β = 0.24; *p* = 0.02) and received interventions addressing treatment in drug effects were significantly associated with reduced needs, measured by the change score of the NEAT subscale *exacerbations* (β = 0.18; *p* = 0. 04).

## Discussion

This study provides initial evidence suggestion the benefits of pulmonary rehabilitation in adults with asthma in relation to health care needs and confirms prior research on asthma control.

Initially, we found that unmet health care needs were reduced during rehabilitation and that this effect was associated with the utilization of suitable asthma interventions in a dose-response fashion. To our knowledge, there exist no studies examining the association between pulmonary rehabilitation and (unmet) health care needs and we therefore provide novel evidence. To gain a better understanding, we additionally calculated subscale specific scores on received asthma education/training, which reflect subscale specific topics of the NEAT. Our results indicate that better physician-patient-communication, e.g. physician made more time available in case of special requests or considered personal circumstances during treatment, but also practical training and information on how to use asthma drugs were associated with less health care needs regarding the NEAT subscale *patient expertise*. These findings suggest that not only a better exchange of information between patient and physician, but also a more appropriate handling of drugs may reduce the need for being an expert for one’s own disease. Furthermore, received interventions addressing topics like information on what to do in case of an asthma attack or practical training of breathing techniques tended to be significantly associated with less health care needs in the NEAT subscale *exacerbations*. Similarly, information on drug effects, side effects and interactions of drugs led to reduced health care needs regarding exacerbations. Thereby, we can assume that not only patient education referring to what to do in case of an exacerbation, but also better knowledge of drug effects may help to reduce needs regarding information on what to do in case of exacerbations. Also, received interventions addressing exacerbations were associated with reduced health care needs in the NEAT subscale drug effects. Thereby we can assume that more skills and knowledge regarding possible asthma attacks may lead to a decrease in health care needs regarding drug effects.

Given the findings from our previous study on psychometric properties [20], we have to conclude that the average improvement of the total NEAT score in the rehabilitation group (mean change score of 2.69 unmet needs) is less than the minimal important change score (MIC = 4 unmet needs) we have estimated before. The MIC refers to the smallest amount of change, which is considered important by patients [27, 28]. However, even if the MIC was not reached, the reduction of needs is still considerable, given the short time period of 23 days (SD = 5.00) on average between baseline and follow-up.

In summary, we can assume that a) interventions during rehabilitation are associated with reduced health care needs in a dose-response fashion and b) very specific treatment elements may reduce specific health care needs during rehabilitation. Compared to the rehabilitation group, we did not find any improvement of health care needs in patients who most likely did not receive any kind of new intervention (control group). By using ANCOVA we were able to ensure that this result is due to participation in rehabilitation and not merely to demographic differences such as age, gender or educational level. Results of the ANCOVA showed that members of the control group had significant more health care needs at follow-up than members of the rehabilitation group. Thus, one can be assumed that unmet health care needs in the rehabilitation group did not decrease merely due to demographic characteristics.

Based on these results, our study should be understood as an initial step to provide evidence. Experimental studies that are based on the randomized allocation of pulmonary rehabilitation and that maximize the likelihood of adequate control groups (i.e. randomized controlled trials, RCTs) are needed to provide more valid insights into the causal relations between rehabilitation and health care needs. Although conducting an RCT on this matter remains challenging, as people with considerable health problems cannot be withheld from rehabilitation.

To our knowledge, there is only one waiting-list RCT of pulmonary rehabilitation in patients with uncontrolled asthma and this RCT demonstrated that rehabilitation is effective, e.g. in terms of asthma control [17]. This study shows that rehabilitation improves asthma control (measured by the ACT) with clinically relevant effect sizes. In the present study, we could confirm these results and found that individuals with asthma who participated in pulmonary rehabilitation displayed significant improvement in terms of asthma control. Whereas mean control was poor at admission, at discharge participants showed an ACT mean score of 20, indicating rather adequate levels of control. While at admission only 35% of participants showed well-controlled asthma, at discharge 56% reported an ACT score of 20 or more. On average, the ACT score improved by 2.29 points, which is slightly less than the identified MIC, which equals 3 points [29]. The slightly more limited improvement of the ACT in this study could be explained by relatively high ACT mean scores at admission (17.39, SD = 4.61). While ACT scores between 5 to 15 indicate a very poorly controlled asthma, scores between 16 to 19 suggest at least a somewhat controlled asthma. This hypothesis is supported by the examination of the characteristics of only those patients who did not display controlled asthma at admission (ACT≤19). In this group, the mean control improved from 14.76 (SD = 3.39, Min = 6, Max = 19) to 18.26 (SD = 4.14, Min = 6, Max = 25) and thus the mean score changed by 3.65 points. This means that this improvement is probably relevant for patients with asthma. If only participants with very poorly controlled asthma at admission (ACT≤15) were included in the analysis, the change was even more pronounced (baseline: mean = 12.00, SD = 2.61; follow-up: mean = 16.81, SD = 4.43; mean change score = 4.88). In addition, we ran an ad hoc univariate ANCOVA (poorly versus well controlled asthma as independent variable and ACT change score as dependent variable). In those analysis, the effect of pulmonary rehabilitation on the follow-up ACT score differed significantly between well-controlled and poorly controlled patients (*F*(1, 88) = 44.65, *p* <  0.001). This finding could indicate that especially the latter could benefit from rehabilitation.

The improvement of asthma control between baseline and follow-up was not observed in adults with asthma who most likely did not receive any kind of new intervention during follow-up period: control remained poor in this sample. Building on this finding, we calculated a univariate ANCOVA (with the covariates age, sex, educational level, number of allergies, mean follow-up period and the respective baseline score), which also showed that members of the control group had significantly less asthma control at follow-up than members of the rehabilitation group. Overall, our results regarding asthma control support previous findings on the impact of pulmonary rehabilitation on patients’ health care [17, 18].

### Strengths and limitations

Importantly, our results are not based on data from an RCT, but we employed a quasi-experimental design. A RCT is considered the gold standard to examine effectiveness of interventions and its defining feature is randomization. Randomization implies that participants are randomly allocated to either the intervention or the control group which should, if the trial is sufficiently large, yield two samples that are comparable in terms of measured and unmeasured confounders [30]. A quasi-experimental design, by contrast, compares two natural groups without random allocation of participants. Thereby, only limited causal conclusions are possible [31, 32]. Our samples did not originate from the same population and demographics were not equivalent. Differences in the mode of surveying (paper-and-pencil during rehabilitation versus mainly online in the control group) may explain these demographic differences. Thus, some distortion even after control for confounding in the analysis (i.e. *residual confounding*) cannot be excluded. Furthermore, unfortunately no information on medication was collected. Therefore, we are unable to examine a possible link between pulmonary rehabilitation and medication (e.g. reduction of doses).

It is reassuring though that there were no baseline differences in terms of our primary outcomes (NEAT and ACT) between rehabilitation and control group. Furthermore, despite using age, gender educational level, number of allergies and follow-up period as covariates, members of the control group had significant more health care needs and less asthma control at follow-up than members of the rehabilitation group. Also, we included individuals from three rehabilitation clinics and thereby collected data in a multicentre design. Thus, we can assume that the improvement of health care needs and asthma control is not limited to an intervention in a specific clinic (i.e. single centre study); rather it seems that rehabilitation by itself and regardless of the clinic or its slightly different style of intervention administration may improve these outcomes.

Unfortunately, we cannot know with certainty that participants in the control group did not receive any new intervention (like elements of a disease management program) during the 31-day-follow-up period. However, since the period was short, we can assume that no rehabilitation took place. Furthermore, unfortunately no information on medication was collected. Therefore , we are unable to examine a possible link between pulmonary rehabilitation and medication (e.g. reduction of dose).

A special strength of both samples is that we attained good response rate at follow-up (rehabilitation: 93%, control: 67%). Furthermore, responders and non-responders provided similar background data at baseline, except for reported allergies in the control group, which were more common in responders (90% vs. 78%, *p* = 0.03). Also, there existed no significant differences between responders and non-responders in both samples regarding NEAT and ACT scores at baseline. In summary, we can assume that major selection bias across the follow-up is rather unlikely.

### Implications

The NEAT has been found to be a reliable and valid instrument to predict treatment satisfaction [20] and therefore may facilitate the planning of interventions at the beginning of rehabilitation according to shared decision-making between patient and physician through the delivery of patient-centered care. Also, one may speculate that administration of our participant-completed questionnaire may increase awareness among patients. Therefore, they may pay greater attention to their unmet health care needs and the fulfillment of these needs during their treatment. Similarly, it could be very helpful to administer the NEAT at the beginning *and* the end of rehabilitation (such as in the present study) to evaluate the interventions and to examine if important cornerstones are met from patients’ point of view.

## Conclusions

This study provides preliminary evidence suggestion of the benefits of pulmonary rehabilitation in adults with asthma in reducing patients’ unmet health care needs and confirms previous findings that rehabilitation may improve asthma control.

## Data Availability

The datasets used and/or analysed during the current study are available from the corresponding author on reasonable request.

## Abbreviations

ACT: Asthma Control Test
COPD: Chronic Obstructive Pulmonary Disease
NEAT: The Patient Needs in Asthma Treatment
PRO: Patient reported outcome
